# The role of governance in implementing sustainable global health interventions: review of health system integration for integrated community case management (iCCM) of childhood illnesses

**DOI:** 10.1136/bmjgh-2020-003257

**Published:** 2021-03-31

**Authors:** Koya C Allen, Kate Whitfield, Regina Rabinovich, Salim Sadruddin

**Affiliations:** 1 Malaria Eradication Scientific Alliance (MESA), Barcelona Institute for Global Health, Hospital Clínic - Universitat de Barcelona, Barcelona, Catalunya, Spain; 2 Malaria Elimination Initiative, Barcelona Institute for Global Health, Hospital Clínic - Universitat de Barcelona, Barcelona, Catalunya, Spain; 3 ExxonMobil Malaria Scholar in Residence, Department of Immunology and Infectious Diseases, Harvard University T H Chan School of Public Health, Boston, Massachusetts, USA; 4 Child Health, MOMENTUM Country and Global Leadership, Washington, DC, USA

**Keywords:** child health, health systems, review, malaria, health services research

## Abstract

Improving health outcomes in countries with the greatest burden of under-5 child mortality requires implementing innovative approaches like integrated community case management (iCCM) to improve coverage and access for hard-to-reach populations. ICCM improves access for hard-to-reach populations by deploying community health workers to manage malaria, diarrhoea and pneumonia. Despite documented impact, challenges remain in programme implementation and sustainability. An analytical review was conducted using evidence from published and grey literature from 2010 to 2019. The goal was to understand the link between governance, policy development and programme sustainability for iCCM. A Governance Analytical Framework revealed thematic challenges and successes for iCCM adaptation to national health systems. Governance in iCCM included the collective problems, actors in coordination and policy-setting, contextual norms and programmatic interactions. Key challenges were country leadership, contextual evidence and information-sharing, dependence on external funding, and disease-specific stovepipes that impede funding and coordination. Countries that tailor and adapt programmes to suit their governance processes and meet their specific needs and capacities are better able to achieve sustainability and impact in iCCM.

**Key questions**

**What is already known?**

- Governance, country leadership and management in global health strategies for child health have notable impact on programme success. Despite countries participating in the integrated community case management (iCCM) approach and developing policies to implement programmes, the resultant iCCM programmes were not always effective or sustainable.

**What are the new findings?**

- There are four key challenges that have impacted iCCM governance processes, programme effectiveness and sustainability. Specifically, lack of country leadership, the need for local contextual evidence on iCCM programmes to tailor country-specific iCCM programmes and information-sharing between implementers, dependence on external funding and disease-specific ‘stovepipes’ or silos that impede funding and coordination of programme activities.

**What do the new findings imply?**

- A strategic approach targeting each of these key challenges will improve governance of iCCM programmes and increase the likelihood for effectiveness and sustainability over time. Improved programmes may then contribute to reaching country goals for addressing burden of childhood diseases.

## Introduction

Global health interventions should align with population needs and the health issues that resonate from resource constraints in health systems, poor access to health services in the population and governance of programmes that address the burden of childhood illnesses. Programmes that manage resource constraints, integrate lessons learnt and adapt to changing infectious disease conditions can offer resiliency and extended capabilities when facing emerging threats such as novel coronavirus (COVID-19), or other challenges that strain health systems and potentially diminish progress in combating persistent disease threats like malaria, pneumonia and diarrhoea. These diseases are leading causes of mortality in children under-5 years of age (U5) and can be better addressed through effective governance of health programmes that facilitate sustainable progress in reducing mortality by improving access to essential health services and implementation of evidence-based interventions with dedicated investment schemes.

Persistent inequities in maternal and child health are enabled by barriers to health coverage, including access to care in urban centres and rural environments. In addition, on access to care, many are not afforded good quality, or face direct and indirect costs for health services that render treatment for preventable diseases improbable. Child health encompasses the nurturing care needed for a child to both ‘survive and thrive’ to their greatest potential and well-being. The ‘good health’ that ensues must be facilitated by equitable access to care.^1 2^ Many communities face persistent poverty due to socioeconomic disparities that require interference through high-level policy development and governmental influence. In these instances, limitations to health intervention impact and low uptake of available health services, then contributes to increased risk for illness, disease-associated morbidity and mortality.^3^ Given the leading causes of mortality U5 can be attributed to three infectious diseases, programmes targeting these diseases can have considerable impact in reducing morbidity and mortality, provided the programmes are governed effectively and offer reach to the underserved communities with the highest burden of disease. As a primary indicator for concern, reductions in mortality would denote progress for goals in child health. The United Nations’ Children’s Fund (UNICEF) global child survival call to action asked countries to strive for 20 or fewer deaths per 1000 live births by 2035,^4^ and their Strategy to Health 2016–2030 emphasises the necessary shift towards a health system strengthening approach that places a focus on equitable access through integrated, and community-based care.^2^ Providing equitable access however requires that the primary health system has the infrastructure and resources to drive successful programme implementation. Moreover, governance, including the coordination, partnerships and management of programmes that sit within broader health systems’ strengthening and global health strategies is intimately linked with sustainability, and anticipated success. From this stance, this project sought to understand governance attributes linked to success of integrated community case management (iCCM) programmes to identify thematic challenges in programme adaptation to national health system (NHS) structures.

### Health systems’ strengthening and governance in iCCM

A strong health system connotes multisectoral engagement and hosts a community-based system for accessing health services at a local level.^3^ ICCM is evidence-based and focuses on improving access to effective case management for malaria, diarrhoea and pneumonia through deployment of community health workers (CHW) to increase reach to underserved populations.^5 6^ The concept of CHW programmes in public health systems is not novel;^7–11^ however, the community-based government-led strategies that utilise them are varied by country with differing levels of success.^12 13^ While evidence has mounted showing the potential impact on child mortality through increased coverage of quality treatment services, challenges remain in achieving the greatest impact from iCCM.^6 14 15^

Since 2010, the iCCM strategy has complemented the WHO Integrated Management of Childhood Illnesses (IMCI) strategy that was initiated in 1999 to focus on delivery of treatment interventions through an integrated case management approach at the health facility level. A joint statement on iCCM by the WHO and the UNICEF, in 2012, stated that delivery of health services is often weak with low coverage for populations that have the greatest need.^5^ While IMCI had many successes, there were clear inefficiencies in reach to the most vulnerable populations, and needed strategic adaptation to extend its reach to address insufficient coverage and capture underserved populations in rural communities with high burden of illness and low utilisation and access to care. In estimates of the potential impact for community case management, significant reductions in morbidity and mortality made an integrated approach plausible.^5 16^ Prompt and effective community management of pneumonia, malaria and diarrhoea has been found to reduce mortality by 70%, 60% and 70%–90%, respectively.^5^ The iCCM programmes use CHWs based in their respective communities to deliver diagnostic and treatment services for multiple childhood illnesses.^10 11^ This includes training, equipping and supporting CHWs to assess, classify and (1) treat uncomplicated diarrhoea, pneumonia and malaria using oral rehydration salts (ORS)/zinc, oral antibiotics and artemisinin-based combination therapy respectively; and, (2) refer children with signs of severe illness and acute malnutrition to an appropriate referral facility.^5 17^

In the context of health system strengthening, iCCM fits as a programmatic contribution to overall goals set forth by WHO and UNICEF. The WHO framework for action towards strengthening health systems to improve health outcomes, addresses challenges to ensuring essential public health functions exist in an effective system that meets population needs.^5^ Similarly, UNICEF includes community health, national investments and governance as core aspects of their solutions for challenges to improving health systems.^3^ ICCM as an intervention begins to fill key gaps in reach and coverage identified in the implementation of the IMCI programme. In that regard, it is imperative to understand what is needed for successful implementation and sustainability of iCCM, as well as lessons learnt from the past implementation failures or scale ups that did not achieve maximum impact.

Governance, country leadership and management in global health strategies for child health have notable impact on programme success. Commitment to corresponding policies has also contributed to the greatest reductions in child mortality.^18^ Approximately, one-third of countries participating in global strategies have developed multisectoral policies related to social determinants of child health;^6^ however, the strategic approaches to meet national goals related to child health needs are often not normalised, lack leadership and the prioritisation that is required to achieve effectiveness and sustainability.^6 19^ For example, in a review of IMCI, 72 of 92 participating countries had an IMCI community health component where CHWs provided treatment for children. Of those 72 programmes, only 52 countries had provisions for iCCM.^20^ Governance is not prescriptive or normative; rather, it is relative to the society, culture, politics and systems at play. Decision-making processes, alongside political systems and social structures exist and influence adoption of global strategies, such as iCCM. Despite global consensus and awareness for the need to apply iCCM for achievement of national child health goals,^5 14 21^ establishment of country-level policy varies.^22^ Where supportive policies do exist, challenges remain, extending from policy to programme implementation and expansion.^23 24^ Understanding the process of policy development, strategic management through programme initiation and implementation is necessary to gauge programme potential for success and sustainability in a country.

## Methods

The purpose of this analysis was to understand governance attributes of iCCM programme success, using iCCM benchmark components^13^ for (a) coordination and policy setting, (b) costing and financing and (c) monitoring and evaluation (M&E) and health information systems (HIS), to identify thematic challenges in iCCM programme adaptation to NHS structures. This was achieved by determining and assessing the technical and financial inputs required for government-led community health systems to provide curative services to their most vulnerable populations and identifying entry-points in the governance process where solutions could be targeted. While much is known about the utility and effectiveness of CHW programmes and community-based case management for childhood illnesses, less is understood about impact of governance on child health initiatives, such as iCCM, as it is adapted in NHS structures.

### Search strategy and selection criteria

A review of indexed and grey literature, including academic publications, organisational reports, government documents, funding and technical support agency evaluations was performed. A search was conducted in the Cochrane review database using the terms ‘integrated community case management’ for identification of registered trials, and systematic reviews. A search was also conducted in the Pubmed central database using the terms ‘integrated community case management’, for which there is no MeSH subheading, [‘integrated community case management’ AND governance], and [‘integrated community case management’ AND policy]. The first tier of selection criteria included articles related to childhood illnesses and/or malaria, and exclusion of studies on case management in the elderly or other special populations (eg, homeless or mental health) or generalised integrated healthcare. References of selected articles were also reviewed for relevance and inclusion. Additional documents were identified on programme websites, specific journal supplements on global health policy, ministry of health websites and funding and technical support organisations resource databases.

### Analytical approach

To investigate iCCM governance, in a broad sense, to improve programme success, the Governance Analytical Framework (GAF)^25^ is applied to iCCM policy development and programme implementation processes. The two basic assumptions of the GAF are that governance processes are found in any society and those processes exist as a set of observable phenomena. As a result, processes can be analysed from a non-normative perspective and governance may be converted into a methodology, for the study of systems of social norms and interactions that determine how public decisions are made.^25^

The lens of key programme attributes for governance; specifically, policy, management and coordination, and financing were used to exemplify systems structures in different countries. In the WHO/UNICEF guidance for implementing iCCM, it was recommended that countries examine policy options, build on existing programmes and initiatives, ensure quality of care, supply-chain management and logistics and monitor and assess data to identify gaps in coverage, patterns in care-seeking behaviour and other key indicators that could be applied to improving programme effectiveness.^5^ As a basis for future programme evaluation and documenting measurable impact, benchmarks for implementation were also developed to facilitate country planning, implementation, monitoring and assessment of iCCM activities. The benchmarks for implementation included: (i) coordination and policy-making; (ii) costing and financing; (iii) human resources; (iv) supply chain management; (v) service delivery and referral; (vi) communication and social mobilisation; (vii) supervision and performance quality assurance and (viii) M&E and HIS. Of these benchmarks for implementation, i, ii and viii served as the proxy measure and contextual focus for development of thematic trends in a GAF for iCCM.

### Patient and public involvement

There was no patient or public involvement in this study. However, dissemination of the study results to relevant actors within iCCM policy development and partnering organisations will impact the wider patient communities reliant on iCCM services. Improved governance of global health programmes will aid in improved access and coordination of services needed to combat childhood illnesses.

## Results

**Figure 1:**
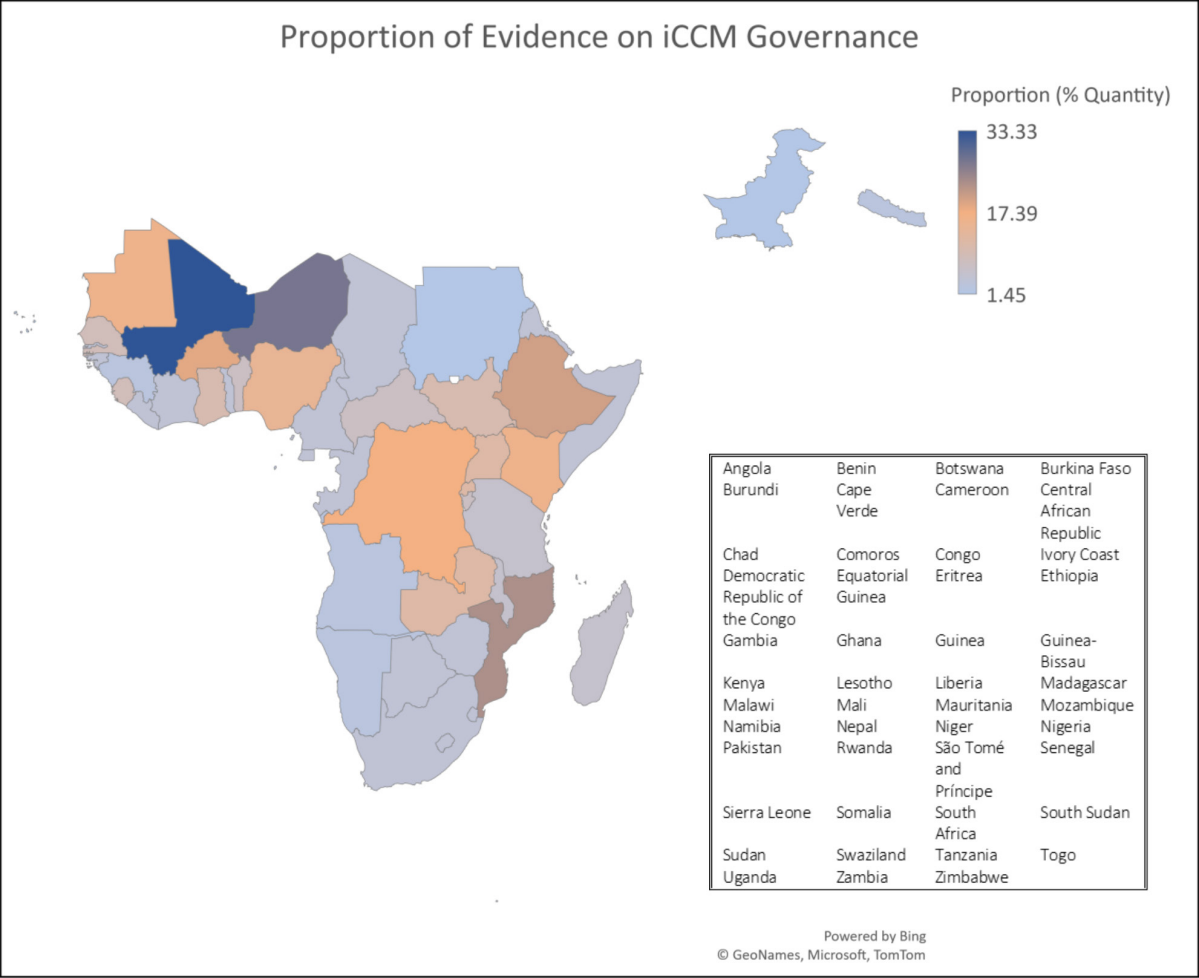
Country-specific evidence for iCCM governance. Countries^47^ with available evidence for iCCM programme implementation. The map and corresponding list of countries depicts the percent quantity or proportion of evidence used to understand governance for iCCM and reflects the potential bias and range of documentation on programme implementation. iCCM, integrated community case management,

A total of 47 countries were included with varying levels of available information on policy and programme uptake. The countries selected were included based on the available evidence identified through the search strategy for countries that have implemented iCCM programmes. The map in figure 1 depicts the percent quantity of evidence by country that was used to understand governance for iCCM. Total evidence by governance attribute was stratified by (1) policy, (2) coordination and management, (3) costing and financing and (4) neutral. Articles that discussed cost or finance in the context of or with policy and sustainability were included in the policy category. Similarly, studies that conducted evaluations were included in the coordination and management category.

### Governance analytical framework for iCCM

**Figure 2:**
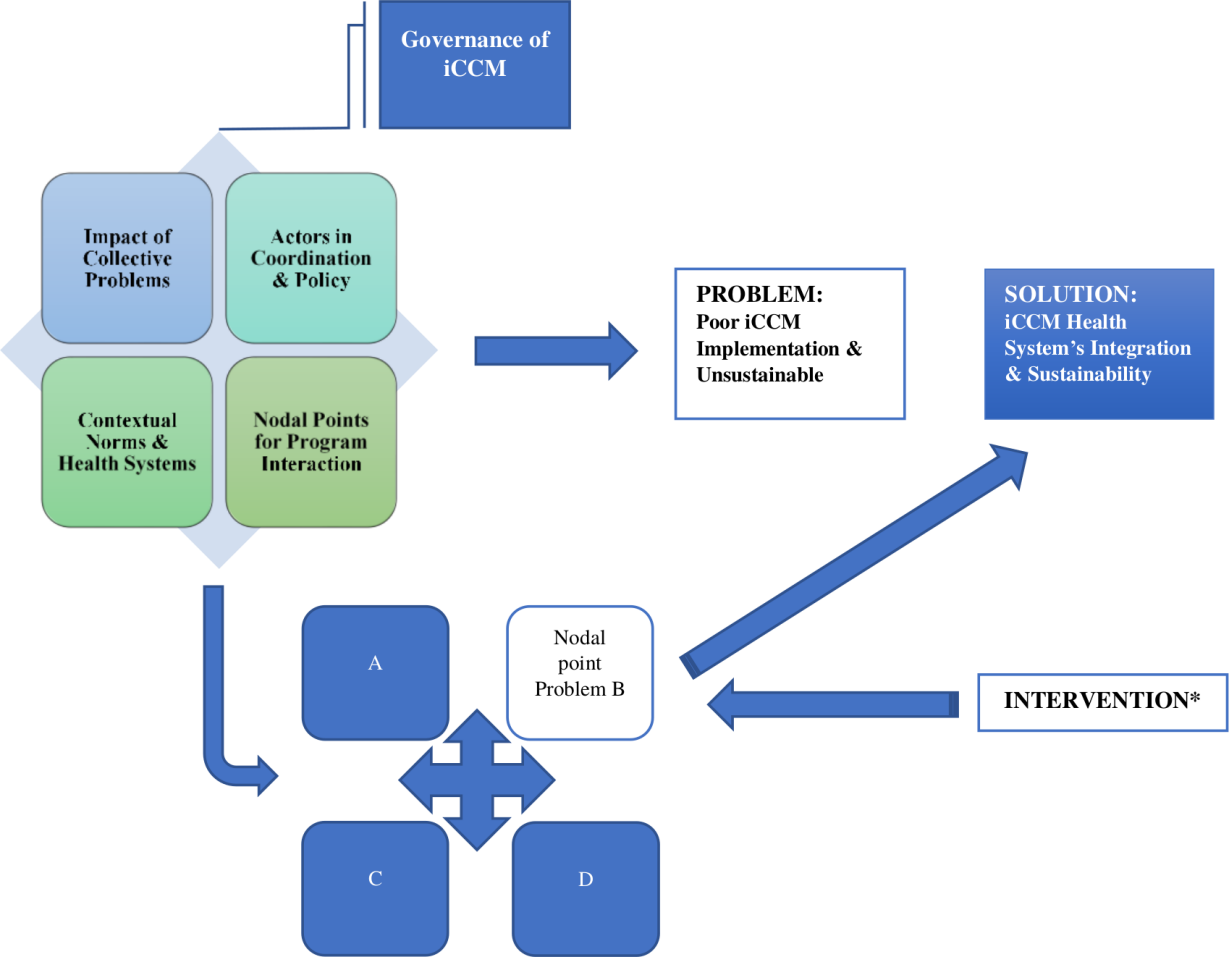
Governance analytical framework (GAF) for integrated community case management (iCCM). Adapted GAF^17^ for analytical interpretation of iCCM governance processes. *Intervention includes efforts to address problems at all nodal point interactions, or key challenges to the iCCM governance structure that can influence a positive outcome and solution for health systems integration and programme sustainability.

The iCCM governance structure was described generically, within the GAF^25^ as (a) the collective problems impacting iCCM success; (b) actors involved in the coordination and policy-setting of iCCM; (c) contextual norms for health systems in a given country and (d) the nodal points that serve as the intersection for programmatic interactions. Figure 2 reveals the iCCM governance structure and illustrates the process for introducing interventions that address nodal point problems to achieve success in iCCM health system integration and sustainability. Using this iCCM governance structure, resultant key themes exemplify significant challenges to governance processes, using the empirical evidence from country-specific examples of iCCM. Each component of iCCM governance is further explained followed by a thematic summary of the key challenges and considerations for addressing them.

#### Problems impacting iCCM success

Countries are often receptive to global health strategies that target key issues relevant to the health of their population. Despite receptivity, the anticipated impact to addressing these health problems is not observed. The analysis revealed that one limiting factor is the hesitancy in policy development and limitations in implementation of corresponding programmes.^13 26 27^ Over the last decade, the number of countries developing iCCM policy has steadily increased.^20 28–30^ However, despite written policy advancing iCCM as a supported approach to child health,^20 23 28^ programmes were not always implemented to scale.^23 24 28^ In some cases, policy was written and codified, but implementation never occurred.^20 23^ As previously mentioned, 52 countries of 100 countries surveyed in the review of IMCI, had plans, policy or CHW programme components and infrastructure for iCCM.^20^ This is a significant increase from previous surveys which reported 28 countries implementing iCCM in 2013,^28^ and 18 countries in 2010.^23^

Another problem impeding iCCM success is the challenge of developing policy based on adaptation of broad-based global health strategies to meet country needs while confined by country capacity.^7 22^ Evidence from case studies investigating iCCM policy development and programme implementation revealed that policy may be established in an ad hoc fashion without an informed and formal process.^22^ For success, translating policy to action can be achieved through ‘championing’ and dedicated leadership by the country,^6 14 18 19 22 31 32^ invested collaborators that can offer both technical and financial support^18 33–36^ and community empowerment with a clear demand for use of services by the community.^12 32 35 37 38^

A key concern identified in the analysis relates to insufficient attention for evidence gathering, synthesis and assessment to ensure gaps do not exist in the integration of new evidence to policy and programmes, as was perceived with the IMCI strategy.^6 18 20 39^ With this awareness, lessons learnt from key IMCI programme challenges should be used to inform governance practices for iCCM. In many ways, iCCM partially fills the gaps in the community component of IMCI that were lost to the focus on training and skill enhancements.^6 18^ Provisions should ensure systematic processes for evidence generation and capture by conducting substantive large-scale country evaluations with funding and implementing partners,^24 40^ and using validated tools for measuring impact.^13 41–43^

In assessing early implementation of iCCM, there were few evaluations available to understand the key challenges and concerns in programme implementation or impediments to success.^44^ This has improved recently with substantial increases in evaluations done to generate country specific, and sometimes district or village specific clarifications for programme successes and failures.^15 24 40 45–55^ M&E of iCCM, and broader strategies influencing child health outcomes, improves adaptation of programme structure and service delivery for populations to achieve the greatest impact from programme potential.^6 18 21 28 30 32 56^ Evaluations can provide revelations in programme effectiveness or the lack thereof. For example, evaluations in Burkina Faso, Ethiopia and Malawi revealed programmatic implementation issues related to coverage, demand and utilisation of iCCM services that led to less than impressive gains in child health targets that could be directly attributed to programme implementation.^45 47 49 57^

The results also show that M&E of programmes can reveal key issues that present opportunities for improving programme management. For example, in recent evaluations of the Rapid Access Expansion (RaCE) iCCM programme, launched in five countries, Democratic Republic of Congo, Malawi, Mozambique, Niger and Nigeria, in 2013 by the WHO, there was an average of 10% reduction in child mortality using the Lives Saved Tool for estimated impact to child mortality across four RaCE project sites. In contrast, the evaluation of the RaCE programme in Mozambique estimated that there were no under-5 lives saved; likely due to broader issues in supply-chain and procurement causing stock-outs of critical medications needed for iCCM success.^15^ Understanding the broader systems needs can aid in programme implementation and eventual impact.^24^ Overall, improved M&E of iCCM programmes from inception can reveal programmatic implementation issues, address needs for coverage, demand and utilisation of services and provide an opportunity to improve on programme management and sustainability.

Finally, a key problem revealed in programme success is that measurable indicators of CHW impact on diseases and child mortality are not always captured or translated to national HISs. The lack of efficient data collection makes key data for measuring programme effectiveness missing in programme evaluations. When useful data is collected, it is often of poor quality and incompatible for comparisons with overarching child health data and targets.^24 43 58^ Efficient and coordinated data generation and surveillance at the local level is critical to inform policy-makers on programme effectiveness, whose support is needed to maintain funding and sustain the programme.^26 59 60^

#### Global actors in coordination and policy-setting of iCCM

The analysis revealed that global actors play a significant role in the governance of iCCM. Each actor brings differences in economic, social, cultural and symbolic capital that influences the mobilisation and support of resources to contribute to policy development, programme implementation and community engagement in a country. The strategic interaction of these actors is inextricably linked with the governance process.^25^ Our results show that in the early assessments of iCCM, success was linked to partnerships in funding, technical support and national governments. There was also evidence that an advantageous approach to adopting iCCM programming was to capitalise on opportunities for health systems strengthening using existing CHW cadres, and once-siloed single disease initiatives.^7 23^

Actors exist at different levels of real or perceived influence and power. Their roles can be classified as strategic, relevant and/or secondary to the use and flow of resources to achieve goals and affect decision-making.^25^ For iCCM, policy transfer and adoption from a global health strategy to a national policy involved different mechanisms. One mechanism included high-level organisations increasing situational awareness by introducing the strategy to countries and transferring policy recommendations. Another mechanism was for funding agencies to present opportunities for financial support to adopt and adapt a particular global health strategy. Organisations and collaborative partners would socialise the strategy in order to set global norms about its effectiveness and utility, and generate interest at a country level.^33^ While this can be construed as positive plays by actors to mobilise others in implementing a global health strategy, in the case of iCCM, there were also alternative dynamics at the country level that could inhibit policy uptake nationally. This puts the top-down and bottom-up approaches to influencing policy adoption at odds. Actors, such as policy entrepreneurs, can influence the policy-making process and help advance or inhibit progress in policy development based on their social or authoritative networks.^61^ These individuals or organisations can become integral in successful advancement of global health strategies.^26 33 34 60–64^

It was also revealed that pre-existing communities specific to disease, organisation and roles also influenced iCCM adoption and uptake at the national level. Silos for funding streams created dissonance in programme prioritisation for specific diseases and encouraged hesitancy in adoption of the iCCM policy with the awareness that dependence on funding would drive imbalanced programme development.^7 33 34^ Some actors, such as The Global Fund, have restructured their funding model to encourage a holistic approach to programme development.^65^ Similarly, the WHO RaCE programme ensured inclusion of sustainability roadmaps and strategies to facilitate increased capacity to manage iCCM programmes.^37^ While it is possible for actors involved in funding mechanisms, or strategy development to alter their line of work to suit necessary changes to drive more comprehensive programming, country-specific political structures may not be capable of restructuring budgetary lines domestically or reorganising programme hierarchical structures that can adequately support generalised comprehensive strategies and funding streams. From this stance, the country context and the actors within are integral in maintaining political will, prioritisation and improving internal collaborations so programmes can be effectively implemented.^27^

#### Country-specific contextual norms

The analysis determined that contextual norms exist at all levels of the governance process, impacting decision-making for conceptual acceptance and policy development to support iCCM. Contextual norms relate to the social norms that exist within the culture and social environment of the country, organisations and communities that play a role in iCCM programme implementation, prioritisation and acceptance. For eventual adaptation to an introduced concept, there lies a process of rejection, resistance and internalisation.^25^ This was evident for early adapters to iCCM policy compared with those countries that exhibited some initial hesitation to implement all iCCM components.^7 26^ As was stated by George and colleagues, ‘Much of the policy resistance to scaling up iCCM is not an aversion to what the intervention promises, but an acknowledgement that the health system effects of iCCM are broad ranging, requiring strategic analysis and resourceful management; skill sets that are under-represented in resource constrained health systems’.^27^ Contextual awareness by national policy-makers on their capacity to implement iCCM impacts policy development and uptake. Additionally, local health system structures need evidence of expansion experiences with iCCM scale-up that reflect their local circumstance. Despite evidence of programme efficacy conceptually, without pilot studies and evaluations in the country context, it was difficult to discern if the same successes and impact to reducing child mortality could be achieved. By highlighting the benefit of these programmes in the local context, actors influence the level of political will, backing and eventual budgeting for implementation and scale-up.

Other necessary considerations of contextual norms became evident in challenges for estimating cost-effectiveness of iCCM implementation based on demand and use by community members.^24 32 36 66^ Health and care-seeking behaviours, as well as understanding the needs and expectations of the populations influenced the use of services and community acceptance, which impacts the effectiveness of the programme.^32 35 38 67 68^ Additionally, global health security is a growing and persistent concern in many areas. Consideration for how to maintain services and advance strategies in environments at risk of local or regional instability, natural disasters, disease epidemics and other emerging threats that affect access and utility of health services is exceedingly important.^69^

#### Nodal points for iCCM interactions

Nodal points are where challenges that impact programme success emerge; key actors are excluded or key populations are not reached through the intervention because of poor considerations for contextual norms. Our results showed that the overall management of iCCM programmes was dependent on adoption of policy into national health strategies. The interactions between policy adoption and eventual programme implementation have a trickle-down effect that influences programme success.^7 32^ For example, stock-outs of critical medications occur as a result of procurement strategies that did not consider iCCM programme needs. At a higher level, technical and bureaucratic considerations lead to concerns in cost and financing for scale-up, or dissonance between political stance and iCCM policy expansion.^26 59 70^ Reluctance to scale-up and expand policy depending on politics^59 61^ can be alleviated by local evidence generation and addressing specific concerns for strengthening key programme elements. Relieving tension at this nodal point facilitates policy development that is compatible with national goals.^23 60^ However, issues can still remain in harmonising programme management and coordination with contextual norms and key actors. Additionally, it was revealed that dependence on external funding, and uncertain outcomes in policy negotiations also impede programme sustainability.^62^

## Discussion

Using the GAF for iCCM, overall problems in iCCM governance can be described within the constructs of the various actors, contextual norms and nodal points that influence policy-making and programme implementation processes. The qualitative analysis revealed thematic challenges that exist within the GAF for iCCM which highlights key issues to address in the coordination and development of iCCM programmes. There are four main themes, described as key challenges to iCCM governance processes; (1) country leadership and integration into NHS’s policy and infrastructure; (2) need for information-sharing and contextual evidence; (3) dependence on external funding impacts sustainability and (4) programme funding and coordination can be limited by disease-specific ‘stovepipes’ or silos. These challenges and relevant considerations for programme planning and implementation are summarised in table 1.

**Table 1:** Addressing thematic challenges in iCCM governance processes

Key challenges	Considerations for programme planning and implementation
Country leadership and health systems’ integration	Support from country leadership and ownership of iCCM concepts to facilitate integration into national health system’s policy and infrastructure
Information-sharing	Ensure information-sharing between country programmes and partnering organisations Increase generation of a contextual pool of iCCM evidence for countries to use
Dependency	Increase national investments to cost and financing to improve sustainability of programmes Reduce dependence on external funding
Stovepipes	Continue efforts to integrate disease specific stovepipes Improve coordination across programme initiatives that encourage a strategic approach to meeting child health goals

iCCM, integrated community case management.

### Country leadership and health systems’ integration

Ownership of iCCM at the country level has been described as a key indicator for programme success across reviews of iCCM programmes.^6 7 18^ The strategy should be integrated as a component to the primary healthcare (PHC) system with clear expectations on objectives and scope of the programme. Notably, iCCM cannot replace the PHC system; however, iCCM can play a significant role in extending reach and effectiveness of the PHC by addressing the needs of the population.^7 14^ ICCM can be strategically implemented within a national plan in collaboration with partners that have clear and predefined roles should be tailored to community structure and needs.^12 47 54 66 68 71^ In addition, data integration with national HIS is ideal. Integration would ensure compatibility with health facility data and allow for adequate M&E of programme effectiveness for child health targets. Improved management of data generation could also reduce burden on CHW responsibilities that have little value for programme management or goals.^24 37 72 73^ While programme expansion may incur additional responsibilities on CHWs, it is important to ensure scale up does not increase burden for workers that will correlate to poor data quality and decreased programme impact.^22 74 75^ For example, in a review of six countries with iCCM policy, CHWs had responsibilities beyond iCCM priority diseases to provide additional child health and even some adult services.^22^ There was evidence of subjective hesitancy to iCCM implementation due to awareness that broader health system needs and capacity for long-term effective management of an iCCM programme were lacking.^27 60^ Enhancing or adapting existing systems aided coordination and contributed to programme success versus development of entirely new programmes. In particular, adaptations that are implemented within existing health paradigms ensures that contextual norms specific to that country are not lost.^27 64^

### Information-sharing

Information-sharing and the generation of evidence that supports iCCM scale-up plays a significant role in policy uptake and advancement. The availability of data emphasising the utility, effectiveness and success of iCCM contributes to policy development and inclusion in NHS.^22^ The shared and collective experiences on approaches for maintenance and sustainability of iCCM is needed so programmes can adapt to changing needs. Local evidence alongside evidence from other countries and collaborating partners would offer a myriad of relevant scenarios to understand factors that impact programme success. This should accompany improved M&E schemes, supported by reliable and quality data, shared in accessible platforms among partners.^37 73^ Local evidence is a significant factor in gaining and maintaining iCCM support; however, data and information-sharing with collaborating partners and other countries offers insight into successful strategies for scale-up, noting potential contextual limitations.^27 38^

### Dependency hinders sustainability

One of the greatest challenges to iCCM is that sustainability of programmes is relative to cost and financing.^7 22 28 37^ Programmes are better suited when key programme attributes are funded in full or at least in part by national governments to minimise dependence on external funding mechanisms. Situating iCCM policy within the NHS facilitates longevity and reliable management of iCCM components, but this is not always possible depending on the capacity and structure of the health system. Reliance on external funding in some cases may be needed for programme maintenance, though it may hinder the governance process and can lead to key issues in programme management and long-term sustainability. For example, in the RaCE Mozambique programme, shortages of supplies and ‘widespread stockouts due to weaknesses in the health system’ limited delivery of supplies and subsequently treatments that resulted in low impacts to child mortality despite a mature iCCM programme and corresponding policy.^15^ Long-term sustainability requires active engagement between the political leaders, organisational partners and other key actors that play a role in programme maintenance. Sustainability planning with external funding and implementing partners should address programme maintenance costs, community-level service delivery platforms, reliable drug supplies and CHW programme funding.^7 22^

### Disease-specific stovepipes

There have been substantial increases in empirical evidence on community-based programmes and CHWs, including iCCM; however, a disease or programme-specific orientation was also evident which hinders the effectiveness of an overall integrated strategy when single disease initiatives hold the foundation of the programme. This raises concerns for the design and sustainability of integrated national programmes.^68^ Dissolution of the disease specific lens can eliminate barriers to comprehensive programming linked to funding requirements. The integrated approach of iCCM uses improved coordination of efforts from service delivery to managerial government ministries and implementing partners at all levels of programming and funding, so there is a greater chance for comprehensive governance practices that support iCCM advancement.

### Limitations

This review was conducted to understand iCCM governance, so there exists some bias in scope based on inclusion and exclusion criteria and use of publicly available information. There are likely publications and reports not included within the pool of evidence used. Moreover, it is evident that bias exists in the literature for country-specific programmes based on funding and implementing partners that could support published documentation of programme governance attributes. As such, there may be country-specific data not included that could have added value to the themes of governance processes identified.

The key challenges discussed are specific to iCCM and are not generalisable to governance of global health programmes in a broad context. There are however some parallels to existing programmes and efforts towards solutions. For example, the high burden, high impact approach is a targeted malaria response in the highest burden countries to drive success in meeting reduction goals.^76^ The approach has succeeded in exemplifying programme progress through high-level political engagement and support. In Uganda, a country-led process of political and multisectoral engagement, and community mobilisation has been established, including increased domestic funding, partnerships within and across government and community programmes and means for M&E. Despite the current success, challenges remain in ensuring sustainability with continued domestic funding, accountability and operationalisation of initiative components to the programme.^76^

## Conclusions

Governance processes for iCCM are influenced by the contextual country norms for health system structure, utilisation and capacity. Moreover, iCCM success is dependent on factors of sustainability, national ownership and evidence-based strategic approaches to implementation and scale-up. A deep understanding of the governance process as it exists within a country facilitates the appropriate adaptation of the iCCM strategy that will suit country’s needs, expectations and capacity.

## Data Availability

All data relevant to the study are included in the article or uploaded as supplementary information.
